# Physiological and Molecular Characterization of the Drought Tolerance-Related QTL qDTY12.1 in *Japonica* Rice

**DOI:** 10.1186/s12284-025-00871-8

**Published:** 2025-11-29

**Authors:** Fan-Yin Tseng, Ming-Hsien Chang, Jen-You Jian, Yu-Chang Tsai

**Affiliations:** 1 Department of Agronomy, National Taiwan University, No. 1, Sec. 4, Roosevelt Rd., Taipei, 10617 Taiwan; 2 Taoyuan District Agricultural Research and Extension Station, Ministry of Agriculture, 139, Sec. 2, Dongfu Rd., Houzhuang Vil. Xinwu Dist., Taoyuan City, 327005 Taiwan

**Keywords:** Drought, Water use efficiency, qDTY, Rice

## Abstract

Drought tolerance in rice is a complex trait regulated by multiple genetic and physiological factors. The quantitative trait locus qDTY12.1 has been associated with improved rice drought resilience, but, the drought tolerance conferred by qDTY12.1 in *japonica* rice remains unexplored. This study investigated the physiological, morphological, and molecular impacts of qDTY12.1 introgression in two rice cultivars, TN11 and TK14. Detached leaf water loss assays revealed that qDTY12.1 introgression lines maintained higher leaf water content than their recurrent parents. Under drought conditions, these lines exhibited moderated reductions in stomatal conductance and stable carbon isotopes discrimination, indicating improved water-use efficiency. Although biomass and yield-related traits were not significantly altered, the TK14_12.1 line consumed less water during vegetative growth. Root phenotyping showed enhanced drought-induced development of thicker and deeper root angles in introgression lines. Biochemically, qDTY12.1 lines accumulated less proline under stress, with distinct expression patterns of proline metabolism and transport genes. Transcriptome analysis under drought and well-watered conditions identified numerous differentially expressed genes (DEGs), including several aquaporins and genes located within the qDTY12.1 chromosomal interval. Notably, the expression of genes potentially involved in stress responses, hormone signaling, and redox regulation was altered in a genotype-dependent manner. These findings suggest that qDTY12.1 modulates complex physiological and transcriptional networks to improve drought adaptation, with variable effects depending on genetic background.

**Supplementary Information:**

The online version contains supplementary material available at 10.1186/s12284-025-00871-8.

## Introduction

In recent years, global climate change has intensified, leading to increasingly severe weather conditions that pose significant challenges to crop growth. Drought, in particular, is a major concern for rice cultivation. Drought affects all stages of rice growth, ultimately resulting in severe yield reduction (Barnabas et al. 2008). Consequently, the development of drought-tolerant varieties and the maintenance of rice yields have been important research objectives for scientists.

Previous studies have demonstrated that the detection of major quantitative trait loci (QTLs) associated with yield under drought conditions, known as Drought yield QTLs (qDTYs), provides an effective approach for breeding drought-tolerant varieties (Kumar et al. 2008). Several qDTYs have been identified from different rice populations. Bernier et al. (2007) discovered the major QTL qDTY_12.1_ in the Vandana/Way Rarem population (Bernier et al. 2007). Ghimire et al. (2012) and Vikram et al. (2011) detected the major QTL qDTY_1.1_ in the Dhagaddeshi/Swarna and N22/MTU1010 populations, respectively (Ghimire et al. 2012; Vikram et al. 2011). Additionally, Venuprasad et al. (2009) identified qDTY_3.1_ and qDTY_2.1_ in the Apo/(2*) Swarna population, while Yadaw et al. (2013) and Saikumar et al. (2014) found qDTY_3.2_ in the IR77298-5-6-18/2* Sabitri and Swarna/WAB 450-I-B-P-157-2-1 populations, respectively (Venuprasad et al. 2009; Yadaw et al. 2013; Saikumar et al. 2014). qDTY_3.2_ was pinpointed as a locus associated with drought-tolerant yield traits in hybrids derived from the local variety Moroberekan and the drought-susceptible variety Swarna (Dixit et al. 2014). Grondin et al. (2018) further investigated qDTY_3.2_, comparing the hybrid progeny BC_2_F_3:4_ with the parental line Swarna (Grondin et al. 2018). They found that under drought conditions, qDTY_3.2_ may mitigate yield loss by promoting early flowering, altering root system architecture to induce deeper root growth, and thereby enhancing water use efficiency.

Among these DTY QTL studies, qDTY12.1 has been consistently demonstrated to contribute to stable drought yield performance across multiple genetic backgrounds. Fine mapping has localized qDTY12.1 to a 1.55 Mb region between 15,848,736 bp and 17,401,530 bp on chromosome 12 (Dixit et al. 2015). Physiologically, the effects of qDTY12.1 vary with drought intensity. qDTY12.1 exhibits significant influence under mild to moderate stress, including increased lateral root growth, enhanced transpiration efficiency, and higher stable carbon isotope discrimination compared with the parental lines (Henry et al. 2014, 2019). These responses are closely linked to modifications in leaf structural traits and aquaporin-mediated water transport. In addition, proteomic analysis indicated that qDTY12.1 regulates pathways involved in nitrogen remobilization, reactive oxygen species (ROS) detoxification, and major metabolic shifts, which collectively enhance lateral root formation, improve source–sink balance, and sustain yield under drought (Raorane et al. 2015b).

Molecular studies have also identified potential functional genes within the qDTY12.1 region. OsNAM (No Apical Meristem) is implicated in root system development, while OsDEC (DECUSSATE) may participate in cytokinin signaling and flowering regulation, both of which influence drought adaptation and yield stability (Sanchez et al. 2022).

When rice encounters drought stress, it reduces stomatal conductance, decreases transpiration, curls its leaves, and limits gas exchange to maintain relative water content, ultimately leading to reduced photosynthetic capacity and lower total biomass (Hussain et al. 2018). Moreover, drought affects root size, growth angle, growth range, and depth. Previous studies have shown that deep-rooted rice varieties exhibit better drought tolerance, thereby maintaining stable yields (Uga et al. 2013). Additionally, research suggests that increasing the number of lateral roots to extend the total root length can enhance drought tolerance in rice (Kano et al. 2011).

To regulate water balance within the plant, plants can adjust growth and development between the shoots and roots through hormonal signaling, such as ABA or ethylene (Sharp 2002), helping to maintain crop growth and yield. Enhancing water use efficiency (WUE) is considered a key trait for sustaining crop production in water-limited environments or under restricted water resources. At the field level, WUE is defined as the ratio of aboveground biomass or economic yield to crop evapotranspiration or total water usage. Physically, it refers to the ratio of net leaf photosynthetic rate to leaf transpiration rate (Bramley et al. 2013).

Yoo et al. found that reducing stomatal density in Arabidopsis could lower transpiration and stomatal conductance while maintaining carbon assimilation, thereby improving water use efficiency (Yoo et al. 2010). In a study by Bramley et al., wheat was observed to absorb water primarily from the root tip region, whereas legumes absorbed water along the entire root system. This suggests that water uptake in wheat is influenced by the number of root branches, while in legumes, it is determined by total root length (Bramley et al. 2009).

Water use efficiency can be evaluated by analyzing carbon isotope discrimination (CID) in plants throughout their growth period. Under well-irrigated conditions, CID is generally positively correlated with yield (Condon et al. 2002). Gao et al. also reported a positive correlation between rice yield and CID, with the strongest relationship observed at the maturity stage (Gao et al. 2018).

Drought tolerance in rice involves complex genetic and physiological mechanisms that regulate plant adaptation under water-deficit conditions. The quantitative trait locus qDTY12.1 has been shown to enhance drought resilience in *indica* rice, yet its functional impact in *japonica* backgrounds remains unclear. We hypothesize that introgression of qDTY12.1 into japonica cultivars improves drought adaptation by modulating physiological traits such as water-use efficiency, stomatal regulation, and root architecture, alongside transcriptional reprogramming of stress-responsive genes. To test this hypothesis, qDTY12.1 from an *indica* donor was introgressed into Taiwan’s major *japonica* rice cultivars, Tainan 11 (TN11) and Taikeng 14 (TK14), and the resulting lines were evaluated for physiological performance, water-use efficiency, and gene expression under control and drought conditions. Elucidating these mechanisms will advance our understanding of qDTY12.1-mediated drought tolerance and support its utilization in breeding climate-resilient *japonica* rice varieties.

## Materials and Methods

### Experimental Materials

The materials used in this experiment were developed through collaboration between the International Rice Research Institute (IRRI), the Taiwan Agricultural Research Institute, the Ministry of Agriculture, and the Taoyuan District Agricultural Research and Extension Station. The donor parents IR743741-46-1-1, with qDTY12.1, were derived from the backcross of Way Rarem/2*IR55419-04 (Mishra et al. 2013; Dixit et al. 2012). The backcrossed inbred lines (BILs) used in this study were derived by the introgression of qDTY12.1 into Tainan 11 (TN11) and Taikeng 14 (TK14) through three rounds of backcrossing (manuscript submitted). Thirteen and four BILs from the BC_3_F_2_ and BC_3_F_5_ generations were selected for the trial evaluation, and the most stable performance BILs from each recurrent parent (TN11 and TK14) used in this study were from the BC_3_F_6_ generation.

### Leaf Water Loss Measurement

Leaf water loss was measured using third-leaf stage rice seedlings grown hydroponically. The tip (3 cm) of the second fully expanded leaf was detached and placed in a growth chamber with light. Each sample consisted of five detached leaves, and each experiment included four biological replicates. Fresh weight was recorded at 0, 2, 4, and 6 h after air exposure. The rate of fresh weight loss was calculated using the formula (FW_0h_ – FW_t_)/FW_0h_. This method assessed the leaf’s water retention ability under drying conditions.

### Water Usage and Evapotranspiration Analysis

Rice seedlings at the third-leaf stage were grown in cylindrical pots (15 cm in diameter and 20 cm in height) filled with soil and water. Each pot was connected at the bottom to a Unicom pipe equipped with a scale to monitor the water level. The change in water level was recorded daily to determine total evapotranspiration. To estimate the seedlings’ transpiration rate, control pots without seedlings were used to measure evaporation, which was then subtracted from the total evapotranspiration. The evaporation amount was then subtracted from the total evapotranspiration. The experiment included four biological replicates per treatment and was conducted in two independent batches, from March to June and from September to January, in a greenhouse. At the end of the experiment, the shoot dry weight was recorded.

### Proline Content Analysis

This method follows the protocol described by Bates et al. (1973). For proline determination, three rice plants at the third-leaf stage were collected after the treatments. Each sample was homogenized and mixed with 3% (w/v) sulfosalicylic acid. After centrifugation at 13,000 rcf at 20 °C for 20 min, the supernatant was combined with equal volumes of Ninhydrin solution and acetic acid. The mixture was then incubated at 100 °C for 1 h, and the reaction was immediately stopped by cooling in an ice bath. Next, toluene was added and thoroughly mixed. The proline content was determined by measuring absorbance at 520 nm.

### Stomatal Conductance and Morphology Analysis

Stomatal conductance was measured using a leaf porometer (Model SC-1, Decagon Devices, Inc., WA, USA) on the most recently fully expanded leaves between 9:00 and 11:00 a.m. under steady light conditions. Measurements were performed during the tillering stage on the same leaves used for morphological analysis. Four biological replicates were included for each line and treatment.

Stomatal morphology was observed between 9:00 a.m. and 12:00 p.m. on clear days during the tillering stage (60 DAT). The lower epidermis of rice leaves was imprinted using transparent nail polish on glass slides. Observations were conducted using an optical microscope (AXIO IMAGER M2, ZEISS, Germany). The number of stomata per unit area and stomatal length were recorded. For each rice line, 20 leaf samples were collected to count the number of stomata, and the data were converted to stomatal density per square millimeter. Additionally, from each of the 20 leaf samples, 10 stomata were randomly selected for length measurement, resulting in a total of 200 stomata measured.

### Stable Carbon Isotope Determination Analysis

During the panicle initiation stage of rice, three fully expanded new leaves were sampled from each plant in the field. The leaves were dried and homogenized using a homogenizer. Analysis of δ^13^C was conducted at the Technology Commons, College of Life Science, National Taiwan University. 2 mg of homogenized powder was packaged into tin capsules and analyzed for stable carbon isotopes using an elemental analyzer coupled with an isotope ratio mass spectrometer (EA-IRMS, Delta V Advantage, Thermo, USA ).

### Root Morphology Analysis

Root traits were evaluated using the basket method originally developed by Oyanagi (1994) and later standardized for rice root phenotyping (Uga et al. 2013; Suresh et al. 2024). The method was adapted in this study using open hemispherical stainless-steel mesh baskets (20 cm in diameter, 10 cm in depth, and 2 mm mesh size) to increase durability under field conditions. The baskets were buried in the field so that their upper edges were level with the soil surface. A four-leaf-stage seedling was transplanted in the center of each basket. For each line, three baskets were used per plot with two replicated plots. During growth, roots penetrated through the mesh, allowing evaluation of root growth direction and distribution. At the sampling stage, the baskets were carefully excavated, and the surrounding soil was gently washed away to retain only the roots that had penetrated the mesh. The number of penetrated roots and their growth angles were recorded in three classes: 0–30°, 30–60°, and 60–90° from the vertical axis, representing shallow, intermediate, and deep root angles, respectively. This modified setup allowed for robust measurement of root system architecture and assessment of drought-induced changes under field conditions. A schematic diagram of the experimental setup is provided in Supplementary Fig. 1.

Roots were then extracted from the stainless-steel mesh, cleaned of excess soil, and laid flat for imaging using a Canon camera (Japan). Root images were processed and analyzed using ImageJ to organize images and measure root angles. Further quantitative analysis was performed with RhizoVision Explorer (Seethepalli et al. 2020) to determine root volume and diameter-based volume distribution. Root diameters were categorized into three classes: <2 mm, 2–5 mm, and > 5 mm, and the relative volume of each diameter class was calculated to characterize root system composition and plasticity under different treatments.

### RNA Extraction

Approximately 0.1 g of rice tissue was collected, and RNA was extracted using the TRI Reagent method (Ambion, US). The tissue samples were homogenized, followed by the addition and thorough mixing of TRI Reagent. After adding chloroform, the mixture was vigorously shaken for 15 s. The samples were then centrifuged, and the supernatant was transferred to a new tube, mixed with an equal volume of pre-chilled isopropanol, and gently shaken. Finally, the samples were centrifuged at 12,000 rcf at 4 °C for 10 min. The RNA pellet was washed with pre-chilled 75% RNase-free ethanol, air-dried after ethanol removal, and resuspended in RNase-free water.

### Reverse Transcriptase Quantitative PCR Analysis

Genomic DNA was removed from RNA samples using the TURBO DNA-free™ Kit (Ambion, US) following the manufacturer’s instructions. cDNA synthesis was performed using SuperScript^®^ IV Reverse Transcriptase (Invitrogen, US) according to the provided protocol. For reverse transcription, DNA-free RNA samples were mixed with 1 µL Oligo dT (50 µM) and 1 µL dNTP (10 µM), incubated at 65 °C for 5 min, and then placed on ice. Reverse Transcriptase Quantitative PCR (RT-qPCR) was conducted using the QuantiNova^®^ SYBR^®^ Green PCR Kit (QIAGEN, Germany) on a QuantStudio 3 Real-Time PCR System (Thermo Fisher Scientific, US). Gene expression levels were normalized using ubiquitin 5 (*OsUBQ5*) as the internal reference gene. The primers used in this study are listed in Supplementary Table 1.

### Transcriptome Analysis

Rice seedlings at the three-leaf stage were subjected to air-drying treatment for 5 h. After treatment, the second fully expanded leaf and root tissues from three biological replicates were collected for RNA extraction. RNA sequencing (RNA-seq) was outsourced to Tools Biotechnology Corporation. The raw sequencing reads were quality-filtered using Trimmomatic to obtain clean reads. These reads were then aligned to the reference genome osa_IRGSP1.0 using HISAT2, and gene expression levels (raw read counts) were quantified using featureCounts. To normalize gene expression, the FPKM (Fragments Per Kilobase of transcript sequence per Million base pairs sequenced) method was applied. Differentially expressed genes (DEGs) were identified based on the criteria of FoldChange > 2 and *p*-adjusted value < 0.05, with normalization and DEG analysis performed using DESeq2. For functional classification, gene ontology (GO) analysis was conducted, and KEGG (Kyoto Encyclopedia of Genes and Genomes) pathway analysis was performed using KEGG Orthology (KO) annotations to identify the biological pathways associated with the analyzed samples.

### Statistical Analysis

Statistical analysis was performed using Microsoft Office Excel. Multiple sample comparisons were conducted using the Student’s t-test or the Fisher Least Significant Difference (LSD) test, with the alpha value set to 0.05.

## Results

### Effects of qDTY12.1 on the Water Loss of TN11 and TK14 Seedlings

**Fig. 1:**
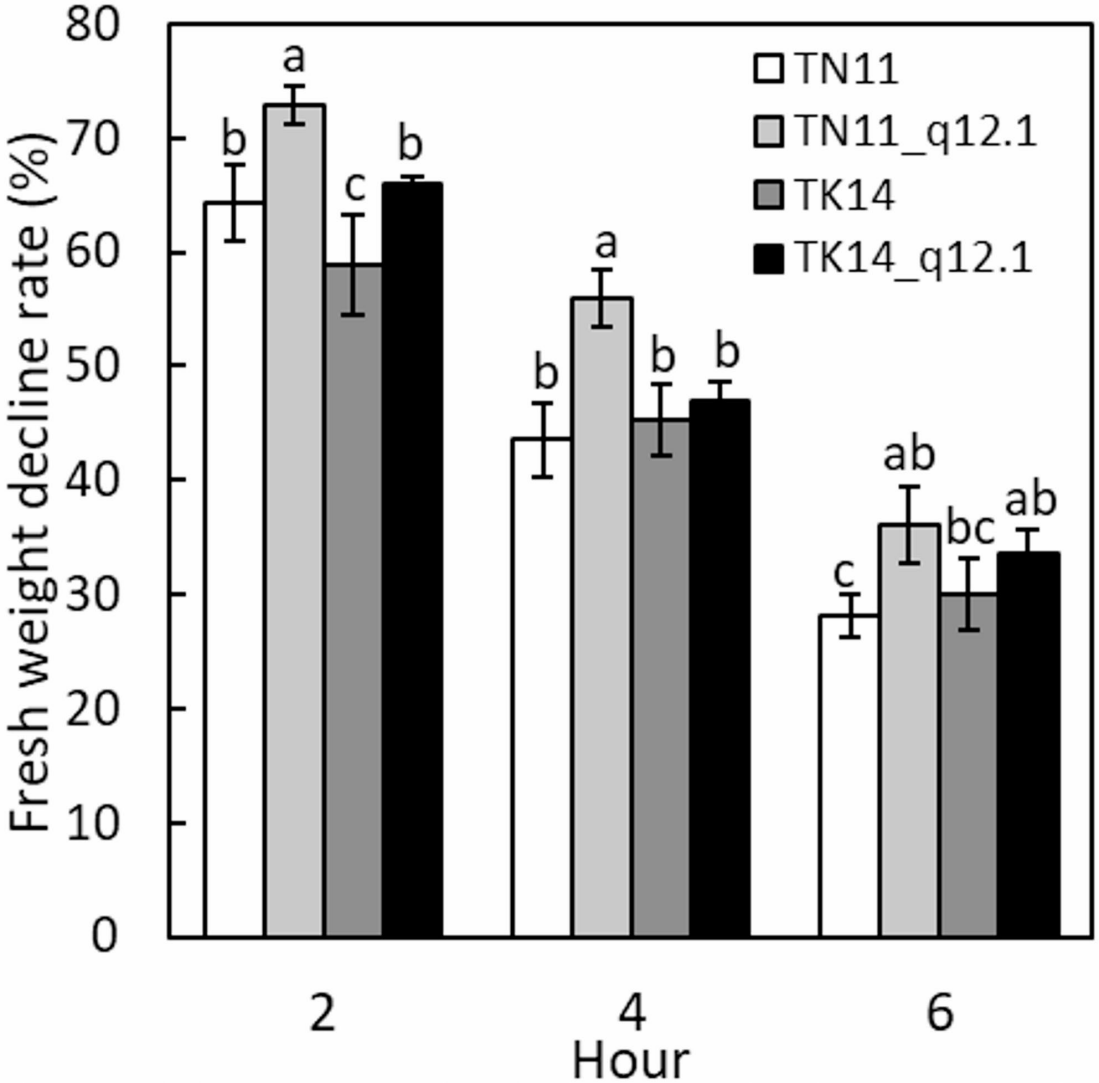
The percentage of fresh weight reduction in detached leaves over the time course. The percentage of fresh weight reduction in the second fully expanded detached leaves of TN11, TK14, and qDTY12.1 introgression lines (TN11_12.1 and TK14_12.1) over time course. Error bars indicate the standard error. Different letters represent significant differences in each time point, ANOVA with LSD test (*P* < 0.05, *n* ≥ 4)

To examine the effect of qDTY12.1 on the leaf water loss, the fresh weight of the detached leaves was measured at 0, 2, 4, and 6 h (Fig. 1). The fresh weight of all samples decreased progressively over time. The percentage decline in fresh weight for TN11, TN111_12.1, TK14, and TK14_12.1 ranged between 59%–73% at 2 h, 44%–56% at 4 h, 28%–36% at 6 h, and 20%–24% at 24 h. Both qDTY12.1 introgression lines exhibited a slower rate of decline in fresh weight compared with their recurrent parents, TN11 and TK14. TN11_12.1 showed the lowest decline rate across all time points. Statistical analysis confirmed that the observed differences in leaf water loss among the introgression lines and parental lines were significant. One-way ANOVA followed by an LSD (Least Significant Difference) test was performed, showing significance at *P* < 0.05 (*n* ≥ 4). These results demonstrate that qDTY12.1 enhances the ability of leaves to retain water under desiccation stress.

### Effects of qDTY12.1 Locus on TN11 and TK14 Water Used Traits

**Fig. 2:**
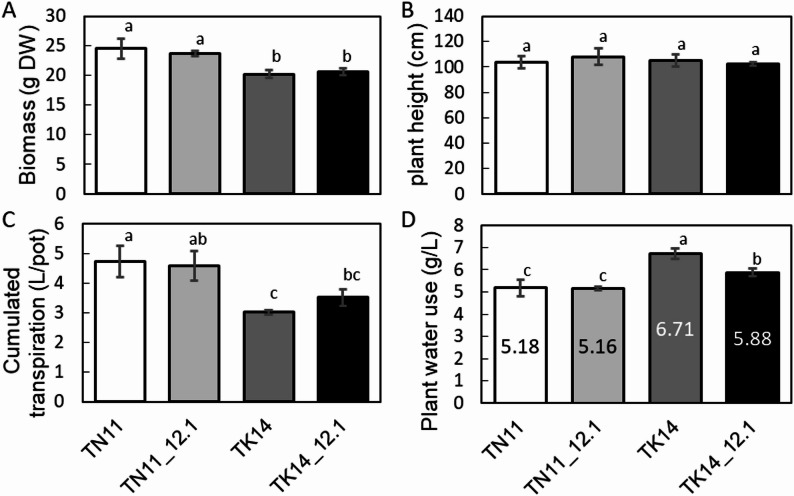
Accumulated transpiration and water usage of TN11, TK14, and the introgression lines at the mature stage.** A** Biomass,** B** plant height,** C** accumulated transpiration of plants per pot, and **D** plant total water use of TN11, TK14, and the introgression lines. Different letters represent significant differences, ANOVA with LSD test (*P* < 0.05, *n* ≥ 3)

To investigate the effect of the qDTY12.1 locus on water use efficiency from vegetative growth to the pre-flowering stage, potted rice plants were used to record cumulative evapotranspiration and biomass (Fig. 2). The transpiration and total water consumption of the plants per pot were recorded daily in the greenhouse. Compared to TK14, TN11 exhibited higher aboveground dry weight and accumulated transpiration, while plant height showed no difference. The qDTY12.1 introgression lines of TN11 and TK14 showed no significant differences in aboveground dry weight, plant height (Fig. 2A and B), or cumulative transpiration (Fig. 2C) compared to their recurrent parents. The introgression of qDTY12.1 reduced water consumption in the TK14 background (Fig. 2D). In contrast, it had no significant effect in the TN11 background.

### Effect of qDTY12.1 on the Transpiration and Water Use Efficiency

**Fig. 3:**
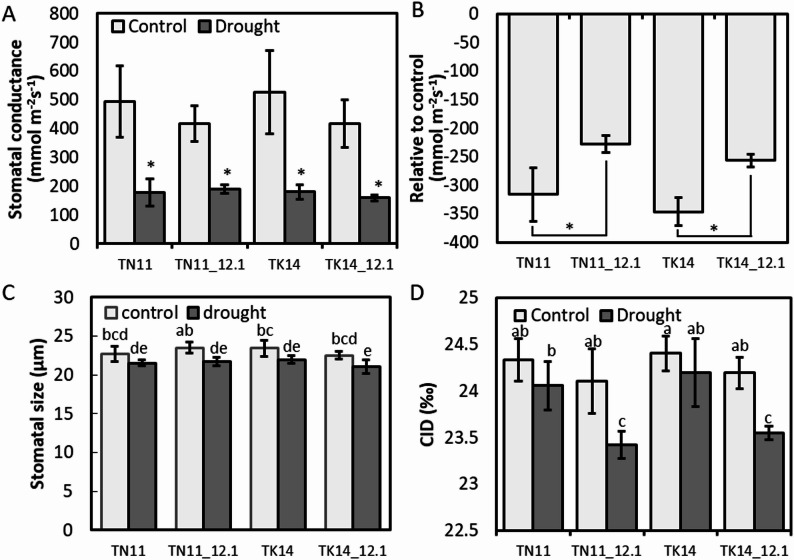
Stomatal conductance (gs) and carbon isotope discrimination (CID) analysis of TN11, TK14, and the introgression lines.** A** The stomatal conductance,** B** relative changes in stomatal conductance,** C** stomatal size, and** D** CID of TN11, TK14, and the introgression lines under control and drought treatment. Deviation =*gs* (drought) - *gs* (control). The asterisks indicate significant differences between parent lines and introgression lines (*n* ≥  3, alpha  = 0.05). Different letters represent significant differences, ANOVA with LSD test (*P* <  0.05, *n* ≥  3)

To further examine the effect of qDTY12.1 on TN11 and TK14, leaf stomatal characteristics during the booting stage were analyzed. Under normal irrigation, the stomatal conductance of both qDTY12.1 introgression lines and their parental controls ranged from 400 to 500 mmol m⁻² s⁻¹ (Fig. 3A). Under drought conditions, stomatal conductance significantly decreased to approximately 200 mmol m⁻² s⁻¹ across all lines. However, the reduction in stomatal conductance caused by drought was less pronounced in the qDTY12.1 introgression lines compared to their parental controls (Fig. 3B). In terms of stomatal density, no significant differences were observed between the qDTY12.1 introgression lines and their controls, with 440–490 stomata per mm² (data not shown). However, stomatal length analysis revealed that under normal conditions, TK14 had a stomatal length of approximately 23.5 μm, which significantly decreased to around 22 μm under drought stress, whereas TN11 showed no significant change (Fig. 3C). Both TN11 and TK14 introgression lines exhibited a significant reduction in stomatal length under drought conditions. Further analysis of stable carbon isotope content in leaves during the tillering stage revealed that TN11 and TK14 exhibited similar stable carbon isotope levels under both normal irrigation and drought conditions (Fig. 3D). In contrast, the qDTY12.1 introgression lines of TN11 and TK14 showed lower stable carbon isotope content under drought conditions compared to their respective parental controls.

### Effects of qDTY12.1 on the Root Morphology of TN11 and TK14

**Fig. 4:**
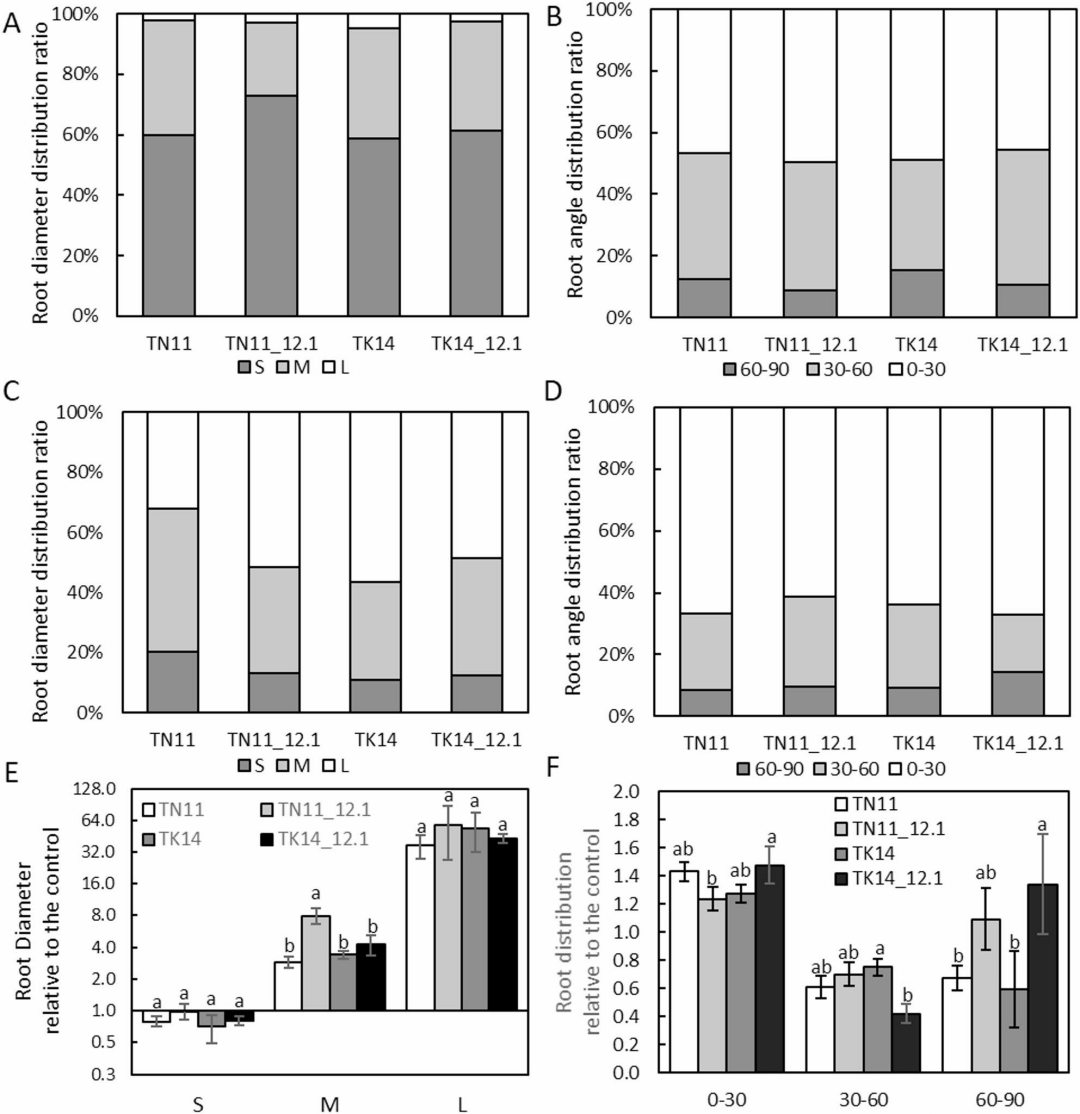
Root morphology analysis of TN11, TK14, and the introgression lines.** A**,** C**,** E** Root diameter distribution ratio and** B**,** D**,** F** root angle distribution ratio of TN11, TK14, and the introgression lines under control (**A**,** B**) and drought (**C**,** D**) treatment at the mature stage. Relative root diameter (**E**) and distribution (**F**) ratio under drought treatment compared to control. Different letters represent significant differences, ANOVA with LSD test (*P* < 0.05, *n* ≥ 3)

Previous studies have shown that qDTY12.1 influences root structure development. Due to the challenges in root phenotyping, this study adapted the iron mesh method—similar in principle to the “basket method” proposed by Oyanagi (1994)—to evaluate root spatial distribution (Fig. 4 and Supplementary Fig. 1). It was found that under normal irrigation, fine roots dominated the root system structure, accounting for approximately 59% and 73% in TN11 and TK14, respectively. In contrast, TN11_12.1 and TK14_12.1 had 60% and 61% fine roots, respectively (Fig. 4A). Root angle distribution showed a similar proportion in the 0–30° and 30–60° intervals, with 45–50% and 36–44% root development, respectively (Fig. 4B). Under drought conditions, coarse root development increased significantly, rising from 6 to 56% in TN11 and from 3 to 52% in TK14 (Fig. 4C). In comparison, TN11_12.1 and TK14_12.1 showed increases from 5 to 43% and from 3 to 49%, respectively. Additionally, root growth angle changed significantly, with a substantial increase in root development within the 0–30° interval, reaching 67% and 61% in TN11 and TN11_12.1, and 62% and 67% in TK14 and TK14_12.1, respectively, while root development in the 30–60° interval decreased to 18–29%, with the most significant reduction observed in TK14_12.1 (Fig. 4D).

### Analysis of Yield Components of qDTY12.1 Introgression in TN11 and TK14

The evaluation of agronomic traits was conducted under both irrigated and drought cultivation conditions (Table 1). The drought experiment began 3 weeks after rice seedling transplantation. The soil moisture content gradually decreased from 45 to 20% over a 30-day period, at which point irrigation was resumed. The control group maintained irrigation with a water level 3–5 cm above the soil surface. Flowering time was recorded for TN11, TN11_12.1, TK14, and TK14_12.1 grown under control and drought conditions (Supplementary Fig. 2). All genotypes flowered between 95 and 105 days after sowing, with no significant differences observed between the introgression lines and their parental lines under the same treatment. For traits such as plant height, tiller number, panicle number, 100-grain weight, yield, and dry weight, there were no significant differences between the qDTY12.1 introgression lines and the control varieties under normal irrigation and drought conditions. Under control conditions, the yield of TN11 and TN11_12.1 was approximately 3.6 tons per hectare, while under drought conditions, the yield decreased to 2.6 and 2.3 tons per hectare, respectively, with no significant differences. Compared to TN11, TK14 had a yield of approximately 2.8 tons per hectare, and TK14_12.1 showed no significant difference under either control or drought conditions.

**Table 1:** Mean values of traits of TN11, TK14, and the introgression lines

Traits	Treatments	Lines			
		TN11	TN11_12.1	TK14	TK14_12.1
Plant height (cm)	Control	129.25	132.25	122.75	121.50
	Drought	119.25	128.25	114.50	111.50
Number of tillers	Control	9.0	8.8	7.5	6.5
	Drought	9.5	9.0	6.5	5.8
Number of panicles	Control	8.3	8.5	7.0	6.0
	Drought	8.8	8.8	6.5	5.3
100-grain weight (g)	Control	2.81	2.80	2.58	2.56
	Drought	2.43	2.45	2.40	2.41
Spikelet fertility	Control	0.85	0.90	0.88	0.91
	Drought	0.85	0.75*	0.70	0.74
Dry weight (g)	Control	66.09	69.56	53.57	47.61
	Drought	58.08	62.76	43.85	37.23
Yield (kg ha^−1^)	Control	3583.12	3656.94	2776.62	2452.01
	Drought	2603.45	2346.82	1852.43	1498.01
Harvest index	Control	0.49	0.47	0.47	0.47
	Drought	0.40	0.34*	0.37	0.36

Mean values with asterisks indicate significant differences between parent lines and introgression lines by a Student’s t-test. P value less than 0.05 shown as *. (*n* ≥ 4, alpha = 0.05)

Additionally, TN11 and TK14 both maintained approximately 90% of grain filling under normal cultivation conditions. Under drought conditions, TN11 retained about 85%, whereas TN11_12.1 significantly decreased to 75%. For TK14 and TK14_12.1, the filled grain percentage dropped to 70–74%. The harvest index was approximately 0.47–0.49 across four varieties under control conditions. Notably, TN11_12.1 showed a significant reduction in the harvest index compared to TN11 under drought conditions.

### Effects of qDTY12.1 Introduction on Plant Proline Contents Under Drought Stress

**Fig. 5:**
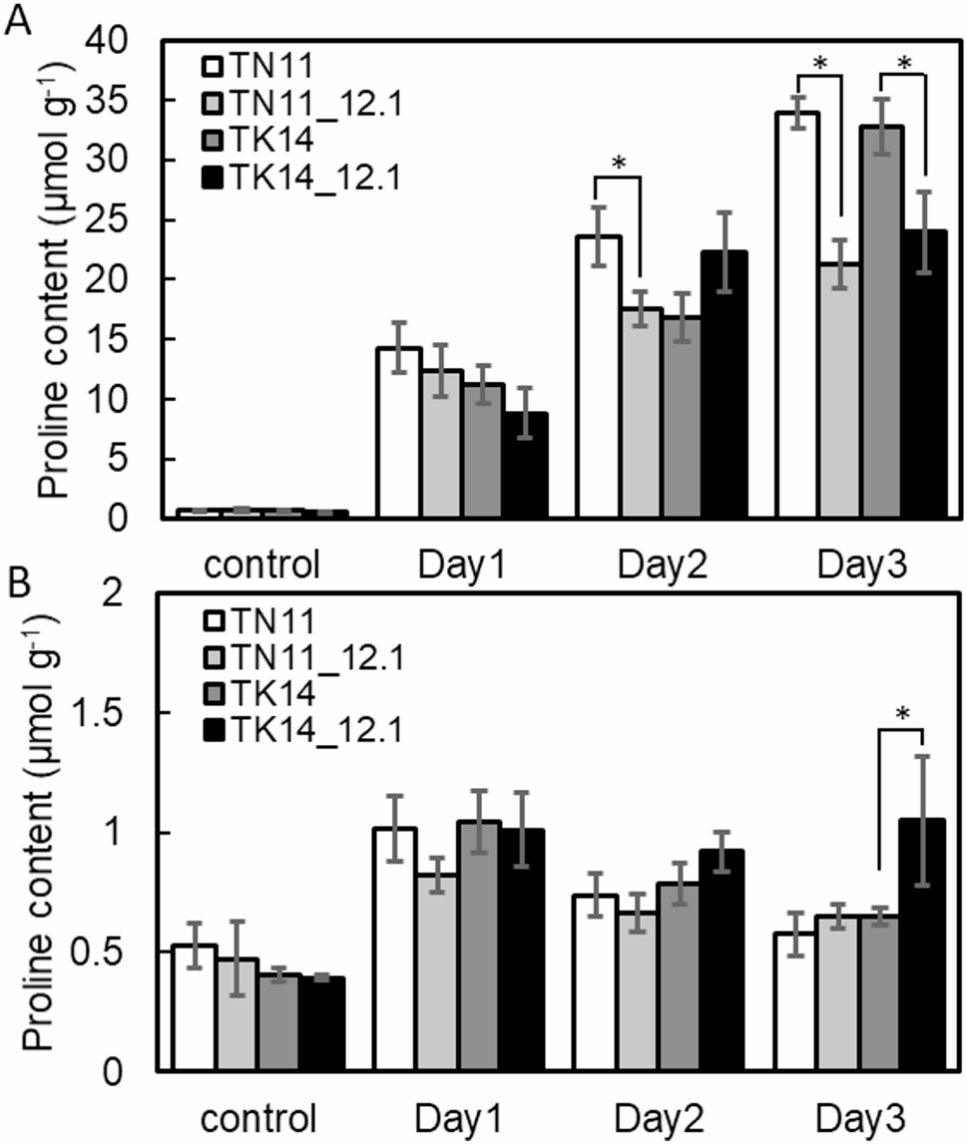
Proline content of TN11, TK14, and the introgression lines in response to 25% PEG treatment. Proline contents in TN11, TK14, and the introgression lines after 25% PEG treatment in leaf tissue (**A**) and root tissue (**B**). The asterisks indicate significant differences between parent lines and introgression lines by a Student’s t-test. P value less than 0.05 is shown as *. (*n* ≥ 3, alpha = 0.05)

To investigate the accumulation of osmolytes under drought stress, proline content was measured in rice seedlings at the third-leaf stage subjected to 25% PEG treatment. The results indicated that on day 0, proline content in the leaves (Fig. 5A) and roots (Fig. 5B) were 0.56 to 0.71 µmol g⁻¹ and 0.39 to 0.53 µmol g⁻¹, respectively, with no significant differences among the lines. Following 25% PEG treatment, the proline content in the leaves of all lines increased significantly from day 1 to day 3 (Fig. 5A). After 2 days of drought treatment, TN11_12.1 accumulated significantly less proline than TN11, while TK14_12.1 showed significantly lower proline content than TK14 after 3 days. Overall, the introgression lines containing qDTY12.1 generally exhibited lower proline content in the leaves than their parental lines. For root tissues (Fig. 5B), a slight increase in proline content was observed in TN11 and TK14 after 1 day of 25% PEG treatment. TN11_12.1 showed no significant difference from TN11. However, on day 3, TK14_12.1 displayed a significantly higher proline content (1.05 µmol g⁻¹) compared to TK14.

**Fig. 6:**
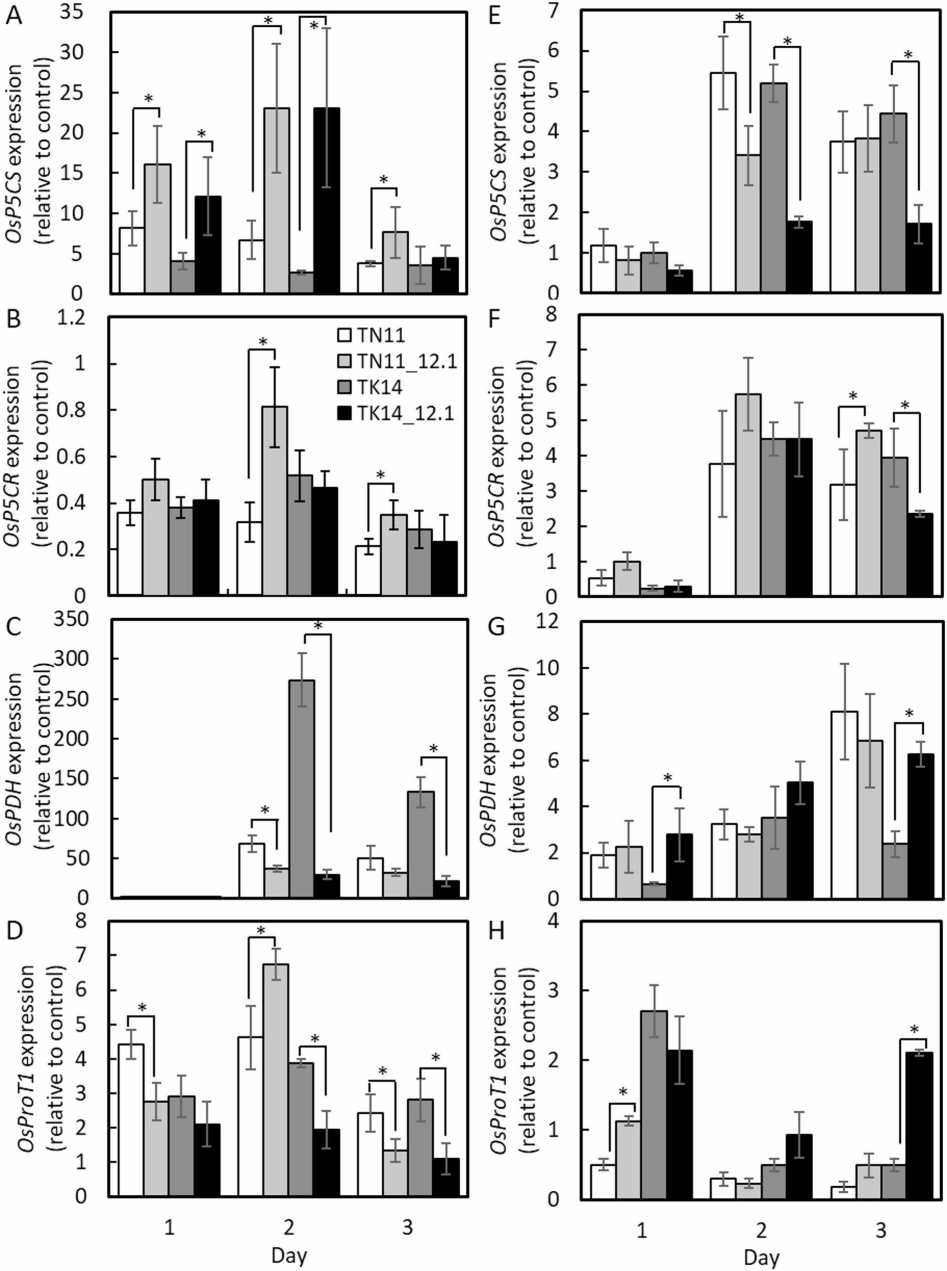
Proline relative gene expression of TN11, TK14, and the introgression lines in response to 25% PEG treatment. The transcription of OsP5CR (Δ’-pyrroline-5-carboxylate reductase) (**A**,** E**), OsP5CS (Δ’-pyrroline-5-carboxylate synthetase) (**B**,** F**), OsProT1 (proline transporter1) (**C**,** G**), and OsProDH (proline dehydrogenase) (**D**,** H**) in third-leaf stage seeding leaves (**A**–**D**) and roots (**E**–**H**) after 25% PEG treatment. The transcript levels were determined using RT-qPCR and expressed relative to the time zero control. The asterisks indicate significant differences between parent lines and introgression lines by a Student’s t-test. P-value less than 0.05 is shown as *. (*n* ≥ 3, alpha = 0.05)

Proline content is regulated through metabolic and transport mechanisms, with key genes such as *P5CS*, *P5CR*, and *PDH* involved in metabolic regulation, while *ProT* influences proline transport. In leaves, 25% PEG treatment for 1 to 3 days significantly induced *OsP5CS* transcription in TN11_12.1 compared to TN11 and in TK14_12.1 compared to TK14 on days 1 and 2 (Fig. 6A). *OsP5CR* expression was significantly higher in TN11_12.1 than in TN11 on days 2 and 3 (Fig. 6B). Conversely, *OsPDH* transcription showed no significant differences on day 1 but was significantly lower in the qDTY12.1 introgression lines than in the control varieties on days 2 and 3 (Fig. 6C). *OsProT1* expression was generally lower in the introgression lines than in their control varieties across most time points, except on day 2, when TN11_12.1 exhibited higher expression than TN11 (Fig. 6D).

In roots, *OsP5CS* expression in TN11_12.1 was significantly lower than in TN11 after 2 days of treatment, while in TK14_12.1, it was lower than in TK14 on both days 2 and 3 (Fig. 6E). For *OsP5CR*, qDTY12.1 introgression resulted in significant transcriptional induction in TN11 and suppression in TK14 after 3 days of treatment (Fig. 6F). *OsPDH* expression was induced only in the TK14 introgression line (Fig. 6G). Meanwhile, *OsProT1* transcriptional expression was significantly induced in the roots of the TN11 introgression line on day 1 and in the TK14 introgression line on day 3 (Fig. 6H).

### Differentially Expressed Genes in qDTY12.1 Introgression Lines Under Drought Stress

**Fig. 7:**
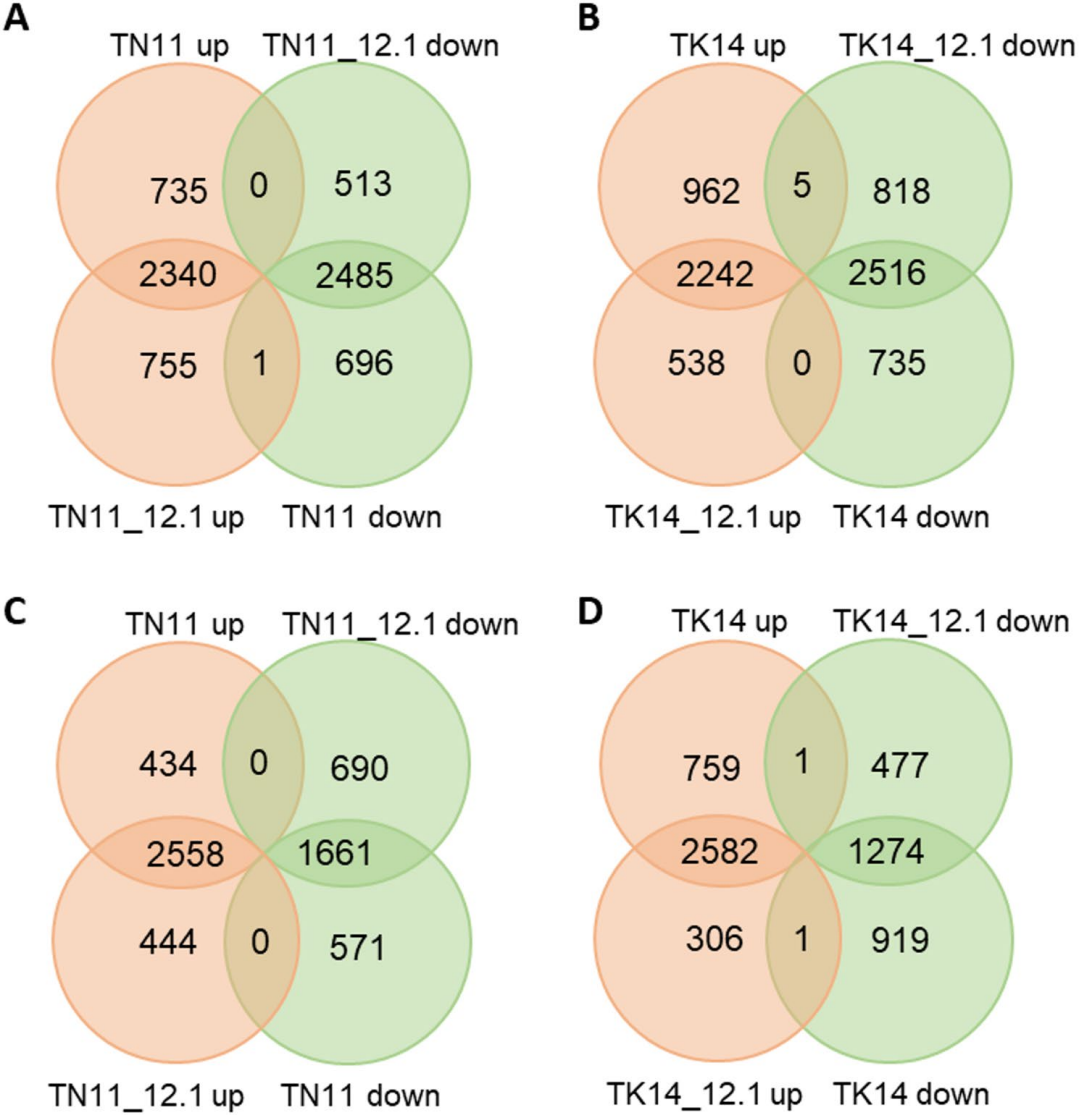
Venn diagram illustrating gene expression differences between qDTY12.1 introgression lines and their parent varieties under drought conditions. Differentially expressed genes (DEGs) were identified in TN11 (**A**,** C**), TK14 (**B**,** D**), and the introgression lines in both leaves (**A**,** B**) and roots (**C**,** D**) following a 5-h air-dry treatment. Genes with a fold change of ≥ 2 and an adjusted p-value (padj) ≤ 0.05 were classified as DEGs

To further compare the molecular regulatory mechanisms of qDTY introgression lines and their parental lines under drought stress, differential gene expression (DEGs) in RNA sequencing was analyzed in both leaf and root tissues after 5 h of air-drying and under normal growth conditions. Under control conditions, the TN11 and TK14 lines with qDTY12.1 introgression exhibited 319 and 955 differentially expressed genes (DEGs), respectively, with expression levels greater than 10 counts and a twofold significant difference compared to their respective parental lines. Under drought conditions, TN11 and TN11_12.1 had 3075 and 3096 DEGs induced compared to the control, respectively, while 3182 and 2998 DEGs were repressed. Among these, 2340 DEGs were induced in both lines, and 2485 were repressed (Fig. 7). Notably, only one gene (*Os01g0611000*) was drought-induced in TN11_12.1 but repressed in TN11. Similarly, in the comparison between TK14 and TK14_12.1, 3209 and 2780 DEGs were induced, while 3339 and 3251 were repressed. Of these, 2242 and 2516 were both induced or repressed, respectively, between the introgression lines and the parental lines. Additionally, five DEGs (*Os01g0937100*, *Os03g0760800*, *Os04g0604300*,* Os04g0686200*, and *Os05g0163300*) were drought-induced in TK14 but repressed in TK14_12.1.

Under drought treatment in root tissues, compared to the control, TN11 and TN11_12.1 exhibited 2992 and 3002 DEGs induced and 3182 and 2351 DEGs repressed, respectively. Among these, 2558 and 1661 DEGs were simultaneously induced and repressed. For TK14 and TK14_12.1, 3343 and 2889 DEGs were induced, while 2194 and 1752 DEGs were repressed, respectively, with 2582 and 1274 DEGs simultaneously induced and repressed. Additionally, one DEG (*Os12g0121200*) was drought-induced in TK14 but repressed in TK14_12.1, while another DEG (*Os02g0787400*) was drought-induced in TK14_12.1 but repressed in TK14.

### Functional Classification of DEGs

In the control treatment, DEGs in leaf tissues of TN11_12.1 compared to TN11 were primarily associated with adaptations to environmental conditions, such as responses to cold, water deprivation, and protein localization maintenance, as classified by Gene Ontology (GO) (Supplementary Fig. 3). Additionally, in the root tissues, the differentially expressed genes between TN11_12.1 and TN11 in the control group were classified in the SCF ubiquitin ligase complex, lignin catabolic/metabolic processes, phenylpropanoid catabolic/metabolic processes.

In the control group, the DEGs between TK14 and TK14_12.1 in leaf tissue were categorized into several GO functional groups, including those related to cell composition and structure, cell surface receptor signaling pathways, cell wall organization or biogenesis, and polysaccharide metabolism (Supplementary Fig. 4). Molecular functions such as hydrolase activity (specifically hydrolyzing O-glycosyl compounds), carbohydrate binding, and signaling receptor activity were also represented. Additionally, KEGG pathway analysis identified four enriched pathways: phenylalanine metabolism, amino sugar and nucleotide sugar metabolism, photosynthesis, and phenylpropanoid biosynthesis.

Under drought treatment, the DEGs in the leaf of TK14 and TK14_12.1 were significantly enriched categories included processes related to the biosynthesis or metabolism of aromatic amino acid families, indole-containing compound biosynthesis, tryptophan biosynthesis or metabolism, and cellular biogenic amine biosynthesis or metabolism (Supplementary Fig. 4). In root tissues, the DEGs between TN11_12.1 and TN11 under drought conditions were associated with the MAPK signaling pathway, plant hormone signal transduction, and plant-pathogen interaction.

### Effect of qDTY12.1 Introgression on Aquaporin Gene Expression

In leaf tissues, 16 aquaporin-related genes were found to be expressed (Supplementary Table 2). For TN11_12.1, two genes (*OsPIP2;2* and *OsNIP2;2*) under control conditions and three genes (*OsPIP2;6*, *OsPIP2;7*, and *OsNIP1;1*) under drought treatment showed significantly higher expression compared to TN11. Meanwhile, one gene each (*OsNIP1;1* under control and *OsTIP3;2* under drought) exhibited significantly lower expression. In TK14_12.1, two genes (*OsPIP2;6* and *OsNIP2;1*) in control and another two (*OsPIP2;6* and *OsNIP2;2*) under drought were more highly expressed than in TK14, while two other genes in each condition showed reduced expression (*OsTIP1;2* and *OsNIP2;2* in control; *OsTIP3;2* and *OsNIP1;1* in drought).

In root tissues, 21 aquaporin genes were detected. TN11_12.1 had four genes (*OsPIP2;4*, *OsPIP2;6*, *OsPIP2;8*, and *OsTIP3;2*) with higher expression than TN11 under control conditions and none under drought. Two genes (*OsPIP2;7* and *OsNIP2;1*) were in control, and one (*OsPIP2;7*) was in drought and showed lower expression. For TK14_12.1, no genes were upregulated in the control, while three (*OsPIP2;8*, *OsNIP2;1*, and *OsNIP2;2*) showed increased expression under drought. One gene each (*OsPIP2;7* and *OsNIP4;1*) had reduced expression in control and drought, respectively.

### DEGs Related To the qDTY12.1 Chromosome Region

**Fig. 8:**
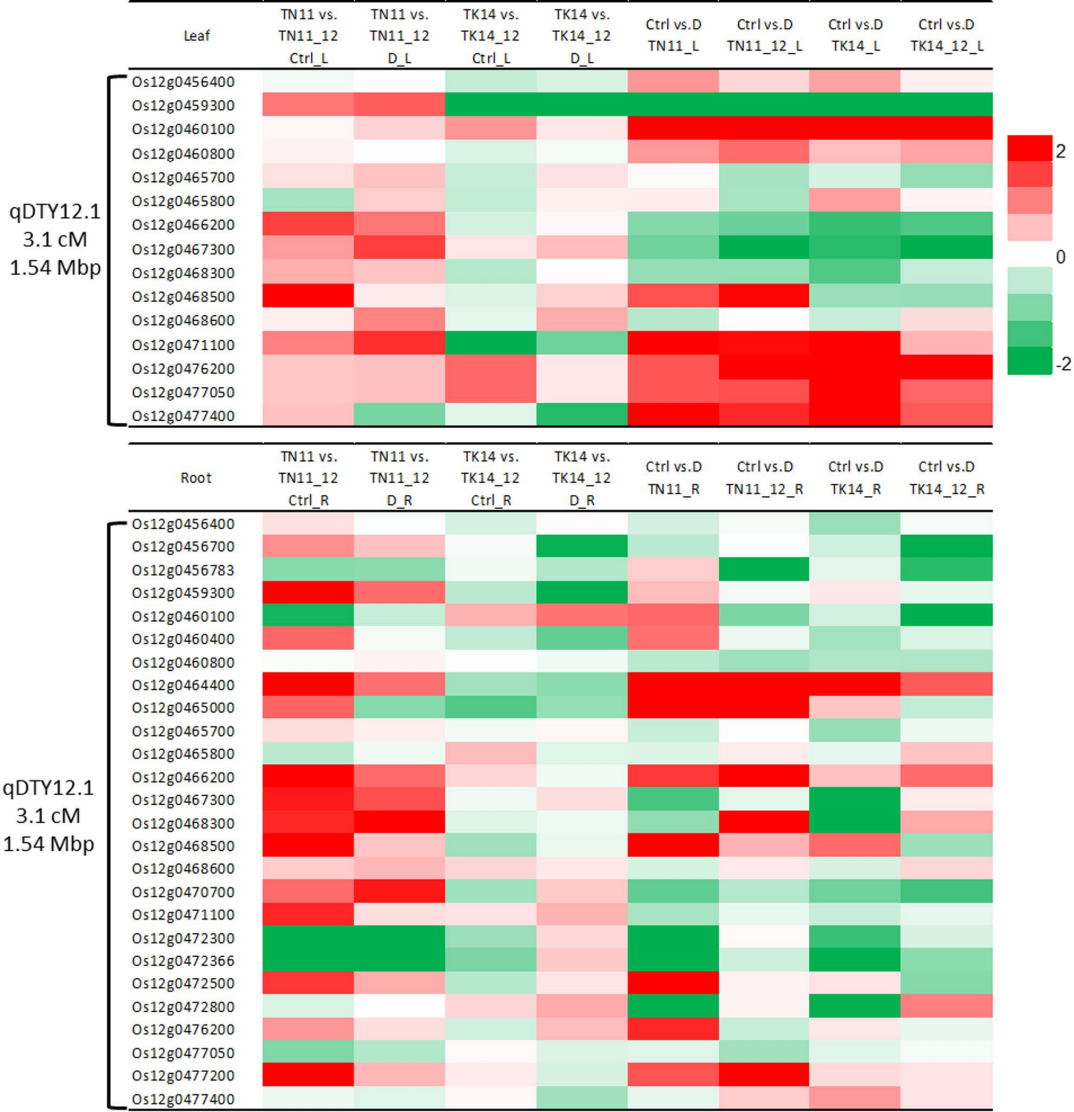
Expression profile of qDTY12.1 region genes in leaf (**A**) and root (**B**) tissues. Heatmap of gene expression and gene description between parents (TN11, TK14) and introgression lines (12.1, TYMK) under different treatments and the gene expression between different treatments in parents and introgression lines were also examined. The difference in gene expression between parents and introgression lines is exhibited by log2 (fold change), LFC. Ctrl: control condition, D: 5 h air-dry treatment

Previous studies had located qDTY12.1 on the long arm of chromosome 12, with the estimated coordinates between 15,848,736 and 17,401,530 bp on the Nipponbare RefSeq (Dixit et al. 2012). Therefore, this study explores the expression of related genes within this region. In this region, 46 genes were identified, of which 19 showed detectable transcriptional expression in the leaves under various treatments (Fig. 8, Supplementary Table 3). For TN11_12.1, nine genes exhibited significantly higher expression than TN11 in both the control and drought treatments. In the drought treatment, one gene showed significantly lower expression. For TK14_12.1, three genes had significantly higher expression than TK14 in the control group and one gene in the drought treatment. Additionally, five and six genes showed significantly lower expression, respectively. In the root tissues, 26 genes exhibited expression. For TN11_12.1, 14 and 7 genes had significantly higher expression than TN11 in the control and drought treatments, respectively, while four and five genes showed significantly lower expression. For TK14_12.1, one and three genes had significantly higher expression than TK14 in the control and drought treatments, respectively, while six and seven genes showed significantly lower expression. Most of these related genes have not been studied in-depth. Through comparison with other plant databases, some genes may be related to disease resistance (*Os12g0460800*, *Os12g0467300*, *Os12g0468300*), carbohydrate transport (*Os12g0476200*), redox enzymes (*Os12g0464400*), and hormone signaling (*Os12g0465700*, *OsDEC*). This suggests that the introgression of qDTY12.1 may influence gene expression within the introduced segment and have varying effects depending on the parental background.

Within the qDTY12.1 locus, *OsNAM* (*Os12g0477400*) and *OsDEC* have been proposed as key regulatory genes associated with drought response. Transcriptome analysis (Supplementary Fig. 5) showed that *OsDEC* expression in roots did not differ significantly among genotypes or between treatments, while in leaves its transcript levels decreased under drought stress. In contrast, *OsNAM* exhibited strong induction in both leaves and roots of TK14 following drought treatment, whereas in TN11, upregulation was observed only in the leaves. Notably, the qDTY12.1 introgression lines showed no pronounced induction of *OsNAM* expression under drought conditions compared with their parental lines.

## Discussion

During rice cultivation, the demand for water resources is becoming increasingly critical, and the impact of climate change has further intensified its effect on food production. Scientists have identified major-effect QTLs associated with drought-related yield using molecular marker techniques (Mishra et al. 2013). QTLs often exhibit varying effects due to genotype-by-environment interactions, which can lead to differences in their efficiency across genetic backgrounds and environmental conditions. Among the QTLs that play a key role in enhancing drought tolerance and yield in rice, qDTY12.1—originally identified from crosses between the *Indica* variety *Vandana* and the upland variety *Way Rarem*—explained the phenotypic variance of 36% and 23.8% under upland and *Indica* rice reproductive-stage drought stress (Mishra et al. 2013; Bernier et al. 2007). Building on these findings, this study aims to evaluate the effects of qDTY12.1 in elite *Japonica* rice varieties, focusing on its potential contributions to drought resistance and yield performance. Identifying QTLs with low environmental interaction effects is advantageous for breeding, as it allows for more efficient trait improvement through marker-assisted selection.

The qDTY12.1 derived from the donor rice IR74371-46-1-1, which was crossed from Vandana/Way Rarem, was introgression into two elite *Japonica* rice varieties, Tainan 11 (TN11) and Taikeng 14 (TK14). Previous studies indicate that TN11 exhibits high yield, broad adaptability, and resistance to blast disease and planthoppers but has poor cold tolerance and low resistance to sheath blight and stem borers (Lin 2005). On the other hand, TK14 is also characterized by high yield and broad adaptability, with better resistance to blast disease, but lower resistance to sheath blight and planthoppers (Chen et al. 1996). Our study found that there were small effects of qDTY12.1 in TN11 and TK14 on the growth and yield under the control treatment (Figs. 2 and 3, and Table 1). However, the accumulated transpiration and the water usage show significantly lower and higher, respectively, in TN11 and TK14 (Fig. 3). These drought-related QTL qDTYs generally have minimal to moderate effects under non-stress and studies found no yield penalty when qDTY12.1 introgressed into both upland and lowland rice (Mishra et al. 2013; Bernier et al. 2009).

Water use efficiency (WUE) refers to the amount of dry matter produced per unit of water used over a given period and is considered one of the key indicators of productivity (Richards et al. 2002). However, assessing WUE is often time-consuming, labor-intensive, and costly. In addition, many measurement and calculation methods are instantaneous and cannot integrate diurnal or daily variations, making them unrepresentative of the overall WUE of the plant (Gulías et al. 2012). Carbon isotope discrimination (CID), which reflects the ratio of assimilation to transpiration during the period of biomass accumulation, is considered a potential approach to overcome these limitations. CID in rice shows a stable correlation with dry biomass, grain yield, and gas exchange parameters, with the strongest correlation between CID and yield observed in samples collected closer to maturity (Gao et al. 2018).

In this study, the water use efficiency was evaluated in both greenhouse and field experiments using cumulated transpiration and CID, respectively (Figs. 2 and 3). In both greenhouse and field conditions, the water use efficiency was comparable between TN11_12.1 and TN11 under normal conditions. Although the TK14_12.1 introgression line demonstrated higher water use efficiency compared to TN11 and TN11_12.1 under greenhouse conditions, its efficiency was still lower than that of TK14. This may be caused by the higher environmental humility and lower transpiration in the greenhouse (Rawson et al. 1977).

In subject to drought conditions, the effects of qDTY12.1 significantly reduced the CID in both TN11 and TK14 at a mature stage, which represents enhancing water use efficiency (Fig. 4). This phenomenon can be correlated to lower relative stomata conductance, which also has been suggested by the effects of qDTY12.1 (Henry et al. 2019). Under drought stress, stomatal conductance declined rapidly as a physiological response to reduce water loss (Wang et al. 2023). Previous studies have demonstrated that low stomatal density is a major determinant of reduced stomatal conductance in drought-tolerant rice genotypes, while genotypes with smaller stomata generally display faster and more sensitive responses to declining water availability (Ouyang et al. 2017). In the present study, the introgression lines TN11_12.1 and TK14_12.1 maintained higher stomatal conductance than their respective parental lines during drought treatment. This result indicates that the qDTY12.1 locus may enhance the ability to sustain stomatal opening and photosynthetic activity under water-limited environments.

Additionally, qDTY12.1 affects root system traits under drought conditions (Suresh et al. 2024). Using the basket method in this study, we observed that qDTY12.1 affects the development of roots with varying diameters and alters root growth angle distribution under drought stress. However, these effects varied depending on the genetic background. In the TN11 background, qDTY12.1 primarily promoted the development of thicker roots, whereas, in the TK14 background, it mainly enhanced the growth of vertically oriented roots under drought conditions. This finding aligns with Clark et al. (2008), who reported that thicker roots penetrate compacted, drought-affected soils more effectively (Clark et al. 2008). Additionally, Yamazaki et al. (1981) found that thicker roots tend to grow vertically—a trend that was also observed in this study (Yamazaki et al. 1981). The influence of qDTY12.1 on root traits has also been demonstrated in previous studies under varying levels of drought stress, showing its most significant effects under mild to moderate drought conditions (Henry et al. 2014). Genotypes with deep, thick roots, more lateral branches, and higher root-to-shoot ratios are often more drought-tolerant (Kim et al. 2020). However, root structure alone may not fully reflect root functionality. Tron et al. (2015) demonstrated that water uptake efficiency depends on rainfall timing: deeper roots are advantageous when rainfall occurs before the growing season, whereas dense shallow roots are more beneficial during in-season rainfall (Tron et al. 2015).

Earlier findings show that introgression of qDTY QTLs improves yield under stress (Mishra et al. 2013; Dixit et al. 2012; Henry et al. 2014; Mohd Ikmal et al. 2019; Suresh et al. 2024). In the present study, field experiments conducted over 2 years showed that under drought conditions, introgression lines in the japonica rice genetic background exhibited yields comparable to their recurrent parent varieties. These differences may be attributed to variations in genetic backgrounds or environmental conditions, as previous studies primarily introduced qDTY12.1 into India or upland rice genotypes.

The differential drought tolerance contributed by qDTY12.1 across genetic backgrounds is also reflected in drought-related metabolic indicators and molecular responses. Previous studies have reported proline accumulation in the roots of drought-tolerant rice varieties (Hien et al. 2003). Previous studies have reported that qDTY12.1 introgression lines accumulate lower proline levels in leaves compared with their parental varieties, *Vandana* and *Way Rarem*, under drought stress, while showing significant changes in amino acid metabolism—particularly serine, glutamate, alanine, and proline pathways—in roots (Raorane et al. 2015a, b). Excessive proline accumulation has also been associated with potential negative effects on yield under prolonged drought stress. In this study, *OsP5CS* and *OsProT1* were both induced in shoots under drought conditions, with higher *OsP5CS* expression and lower *OsProT1* expression in TN11_12.1 compared with TN11. In roots, the opposite trend was observed. The proline degradation gene *OsProDH* was strongly induced in shoots after 2 days of treatment, particularly in TN11, while its induction in roots was less consistent. Although the ratio of *OsP5CS* to *OsProDH* expression was higher in TN11_12.1 shoots, proline content was lower, coinciding with reduced *OsProT1* expression. Furthermore, *OsProT1* has been identified as a key transporter mediating proline and γ-aminobutyric acid (GABA) transport in rice, with differential tissue-specific expression patterns (Lin et al. 2019). This suggests that proline accumulation may be influenced not only by biosynthesis and degradation but also by translocation processes. Previous studies reported variable correlations between proline transporters and proline levels (Ueda et al. 2008; Cha-um et al. 2019). Taken together, these results suggest that moderate proline accumulation and efficient redistribution between organs, rather than excessive accumulation, may contribute to the improved drought adaptation conferred by qDTY12.1.

The transcriptomic comparison between qDTY12.1 introgression lines and their parental cultivars revealed distinct patterns of gene regulation under both control and drought conditions. Under well-watered conditions, a small substantial number of differentially expressed genes (DEGs) were already detected in TN11_12.1 and TK14_12.1 compared with their parental lines. Under well-watered conditions, a substantial number of DEGs were already detected in TN11_12.1 and TK14_12.1 compared with their parental lines, especially enriched within the qDTY12.1 region. Under drought stress, numerous genes were differentially expressed; however, most exhibited similar induction or repression patterns between the qDTY12.1 introgression lines and their parental lines. Combined with the limited number of DEGs observed under control conditions, this suggests that the introgression lines and parental genotypes share a high degree of genetic background similarity. Moreover, these findings are consistent with previous reports indicating that qDTY loci primarily contribute to yield maintenance under mild to moderate drought conditions (Dixit et al. 2014).

Furthermore, transcriptome Gene Ontology (GO) analysis revealed that drought-responsive DEGs in the leaves of TK14 and TK14_12.1 were significantly enriched in pathways related to amino acid biosynthesis and metabolism. The most prominent GO categories included the biosynthetic and metabolic processes of the aromatic amino acid family. These pathways are involved in the synthesis and turnover of amino acids and amine compounds that function as precursors for protein and hormone synthesis, antioxidants, and signal molecules. Regulation of amino acid metabolism has been shown to enhance plant adaptation to drought by maintaining osmotic balance, redox homeostasis, and stress signaling efficiency (Raorane et al. 2015b). These physiological results suggest that the introduction of qDTY12.1 into different *Japonica* rice varieties leads to significant variations in its effects. However, yield performance under drought conditions did not show a significant improvement, suggesting that the influence of qDTY12.1 may weaken or become more variable when introduced into japonica rice.

To understand the mechanism by which qDTY12.1 affects plant growth, researchers have conducted fine mapping on chromosome 12, narrowing its location to 15,848,736 bp to 17,401,530 bp in the Nipponbare RefSeq (Dixit et al. 2012). Within this region, *OsNAM*_*12.1*_ (*Os12g0477400*) has been identified as a potential functional gene of qDTY12.1, playing a crucial role in root development and structural regulation (Dixit et al. 2015). Additionally, *OsDEC* (*Os12g0465700*) may also contribute to qDTY12.1 function, participating in cytokinin signaling pathways and flowering regulation under drought conditions (Sanchez et al. 2022). In this study, transcriptome analysis was used to identify expressed genes within this segment (Fig. 7, Supplementary Fig. 5). The downregulation of *OsDEC* in leaves under drought may indicate a reduction in developmental signaling or cell division processes, consistent with its putative function in controlling organ differentiation. In contrast, *OsNAM*, a NAC-domain transcription factor known to participate in stress-responsive gene regulation and senescence-associated pathways, was strongly induced in the drought-sensitive parental line TK14, implying a stress-responsive activation rather than a constitutive tolerance mechanism. The limited induction of *OsNAM* in the qDTY12.1 introgression lines suggests that qDTY12.1 may confer drought adaptation through alternative regulatory networks that maintain physiological stability without excessive stress signaling. In addition, transcriptional analysis of other genes within the qDTY12.1 locus revealed that TN11_12.1 exhibited markedly different expression patterns compared with TN11, with notably higher transcript levels in roots than in shoots. In contrast, the differences between TK14_12.1 and TK14 were less pronounced, and in some cases, their expression trends were even opposite to those observed in the TN11 background. These results suggest that the regulatory influence of qDTY12.1 on genes within its locus is genotype-dependent and may exert a stronger effect in root tissues than in shoots.

Transcriptome analysis also revealed that qDTY12.1 influences osmotic regulation mechanisms. In addition to previously observed changes in proline accumulation, several aquaporin genes involved in maintaining osmotic adjustment were significantly downregulated under drought conditions. Since aquaporin activity affects transpiration rates and contributes to drought tolerance, their regulation is critical (Lian et al. 2004; Shekoofa and Sinclair 2018; Shivaraj et al. 2021; Sakurai et al. 2005). In the qDTY12.1 introgression lines, the aquaporin genes—*OsTIP3;2* and *OsNIP1;1* in leaves, as well as *OsPIP2;7*, *OsTIP1;2*, and *OsNIP1;1* in roots—were notably upregulated under drought stress, suggesting a potential role in enhancing water transport and stress resilience. Among aquaporins, *OsPIP*s and *OsNIP*s were generally suppressed by drought, whereas *OsTIP*s exhibited increased expression in roots but decreased in shoots, except for *OsTIP3;2*. Previous studies have suggested that PIPs and TIPs are involved in H₂O₂ transport, with TIPs playing a role in reducing cytoplasmic reactive oxygen species (ROS) by transporting H₂O₂ into vacuoles for detoxification, thereby enhancing drought tolerance (Wang et al. 2014). It has been shown that OsPIP2;7 plays a key role in facilitating rapid water movement and maintaining cellular water homeostasis. (Li et al. 2008). This study suggests that qDTY12.1 may influence aquaporin gene expression, but further research is needed to clarify the functional roles of different aquaporins and their relationship to drought tolerance.

## Conclusions

This study demonstrates that the introgression of qDTY12.1 into *japonica* rice backgrounds confers partial improvement in drought tolerance, particularly through enhanced regulation of stomatal conductance and development of the root system. These physiological advantages indicate that qDTY12.1 contributes to maintaining water use and growth under limited water availability. However, the magnitude of its effect in *japonica* backgrounds appears to be less pronounced than that reported in *aus* or *indica* rice, suggesting possible genetic background interactions influencing its expression and functional efficiency.

Future studies should evaluate the effects of other qDTY loci (e.g., qDTY1.1, qDTY2.1, qDTY3.1) in *japonica* rice to identify complementary QTLs that can be combined through marker-assisted pyramiding to enhance yield stability under drought stress. Together, these findings provide a foundation for the development of drought-resilient *japonica* cultivars, supporting sustainable rice production in regions increasingly affected by water scarcity and climate variability.

## Supplementary Information

Below is the link to the electronic supplementary material.

Supplementary Material 1.

Supplementary Material 2.

Supplementary Material 3.

## Data Availability

All data generated and analyzed during this study are included in the main article and its supplementary information files. The raw data supporting the conclusions of this study are available from the corresponding author upon reasonable request.
