# Supplementary material for: Physiological and Molecular Characterization of the Drought Tolerance-Related QTL qDTY12.1 in Japonica Rice
Source: Rice (N Y). 2025 Nov 29;19:1. doi: 10.1186/s12284-025-00871-8 (PMC12770149; doi:10.1186/s12284-025-00871-8)
Supplement: Supplementary file 1 — Supplementary Material 1. [file 12284_2025_871_MOESM1_ESM.docx]

Supplementary Table 1. Primer list.

| Primer | Sequence (5’ to 3’) |
| --- | --- |
| OsP5CR_qF | ATCTCTGTGAAGCCGCAGAT |
| OsP5CR_qR | CAACAGCTGAAGGGGTGTTT |
| OsP5CS1_qF | CAGTTTAGCAGGACTGTTGG |
| OsP5CS1_qR | TGTCATGCCTCCCTACCTA |
| OsProT1_qF | AGGGAGATGGAACAGGATTTG |
| OsProT1_qR | GTCCGCGAAGAAATGGTAGA |
| OsProDH_qF | GTGAGCAAGTACCTGCCGT |
| OsProDH_qR | TCACTCACGTCCCAGCATTG |


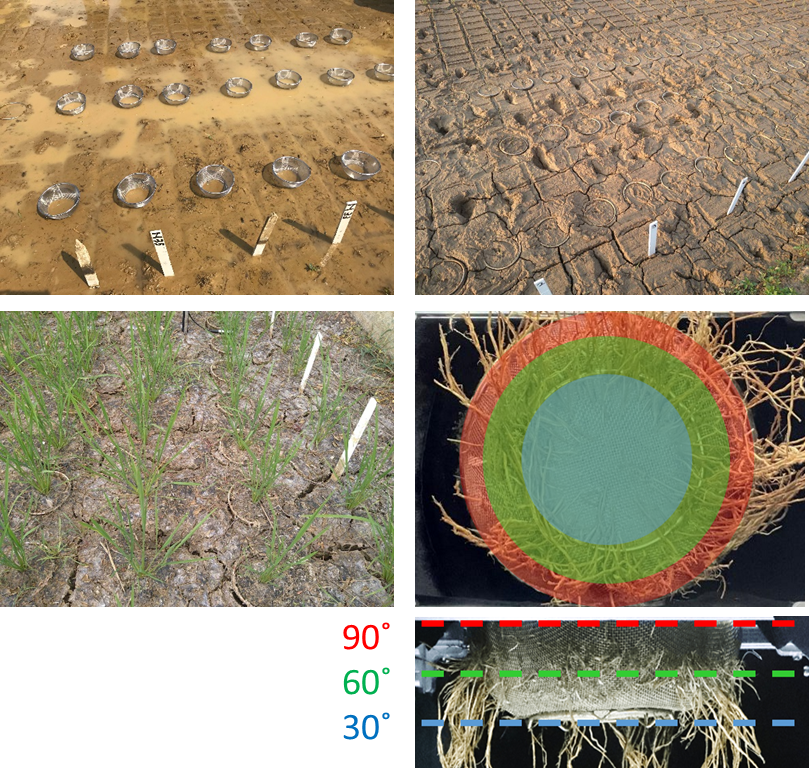


Supplementary Figure 1. Setup for Root Morphology Analysis in the Experimental Field.


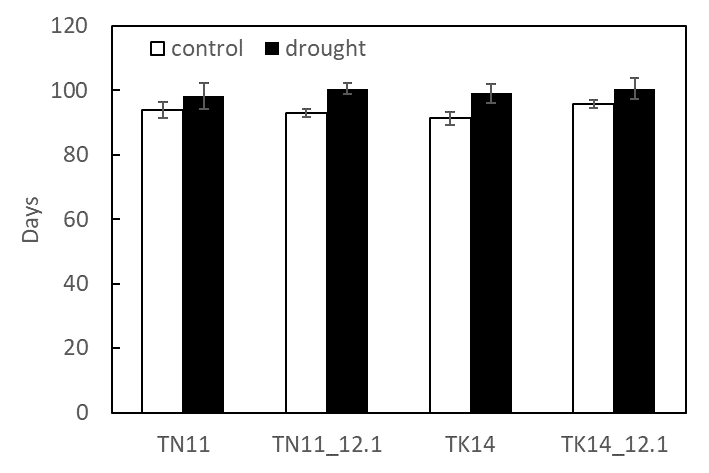


Supplementary Figure 2 Flowering time of TN11, TN11_12.1, TK14, and TK14_12.1 under control and drought conditions. Days to flowering were recorded for four rice genotypes grown under well-watered (control) and drought-stressed conditions. Bars represent mean ± SE (n ≥ 4).


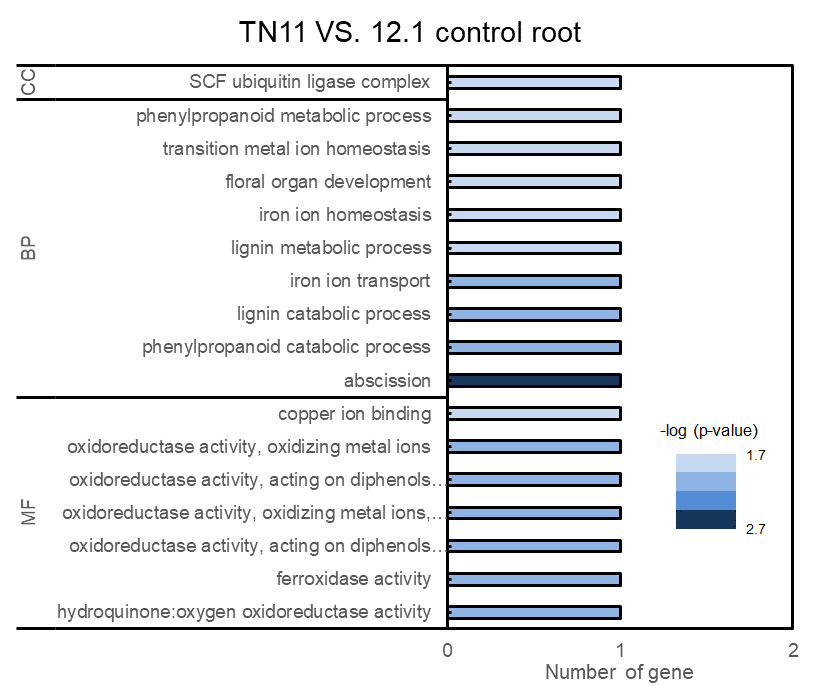

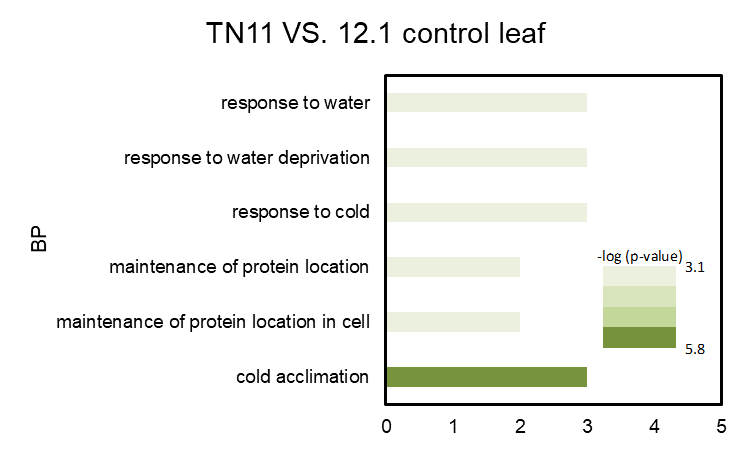


(B)

(A)

Supplementary Figure 3. Gene ontology categorization of DEGs between TN11 and TN11_12.1 under control conditions in leaf (A) and root (B) tissue. Genes with fold change ≥ 2 were defined as differentially expressed genes (DEGs) between TN11 and TN11_12.1 under control condition in leaf tissue.


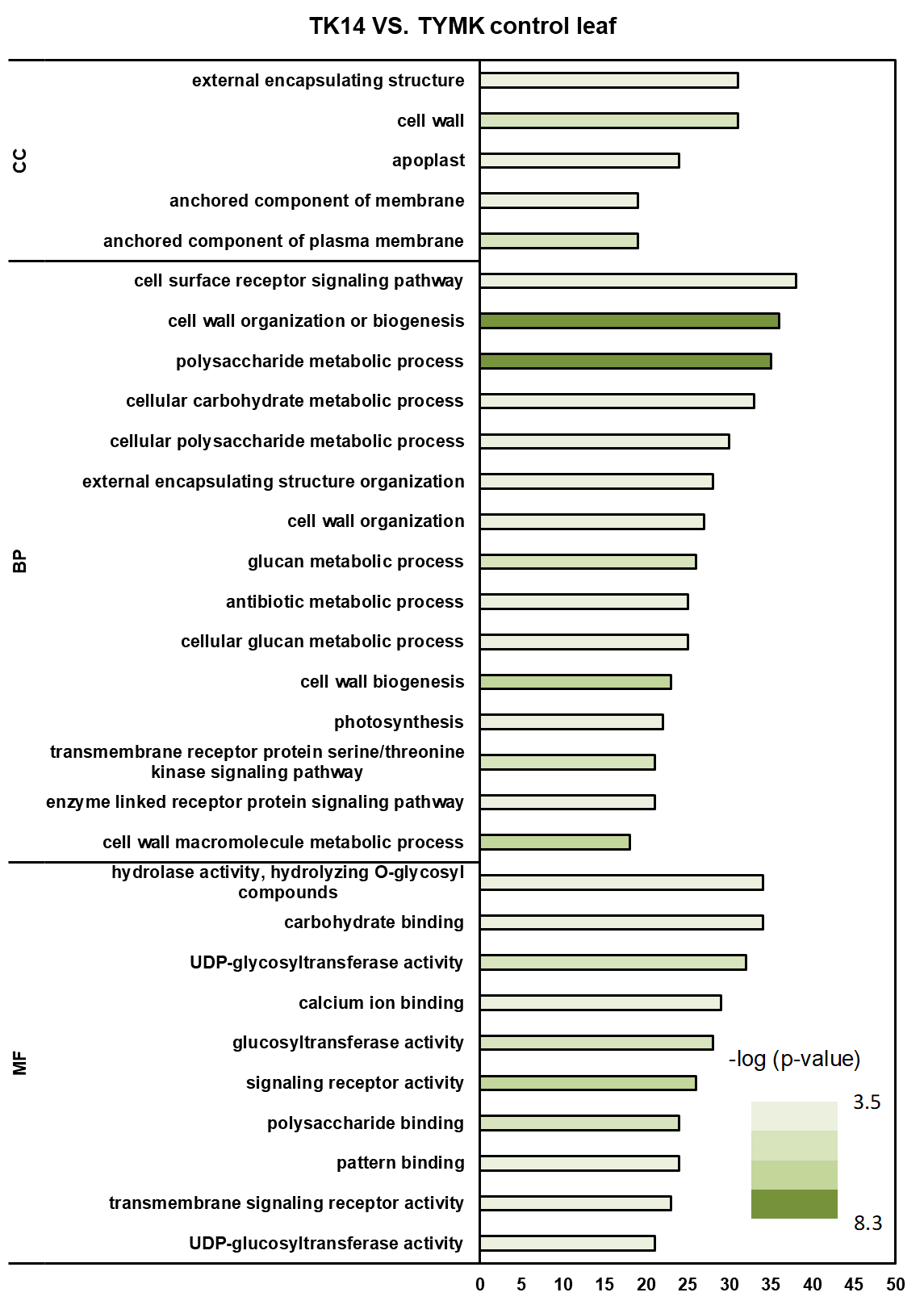


(A)

(Continued)


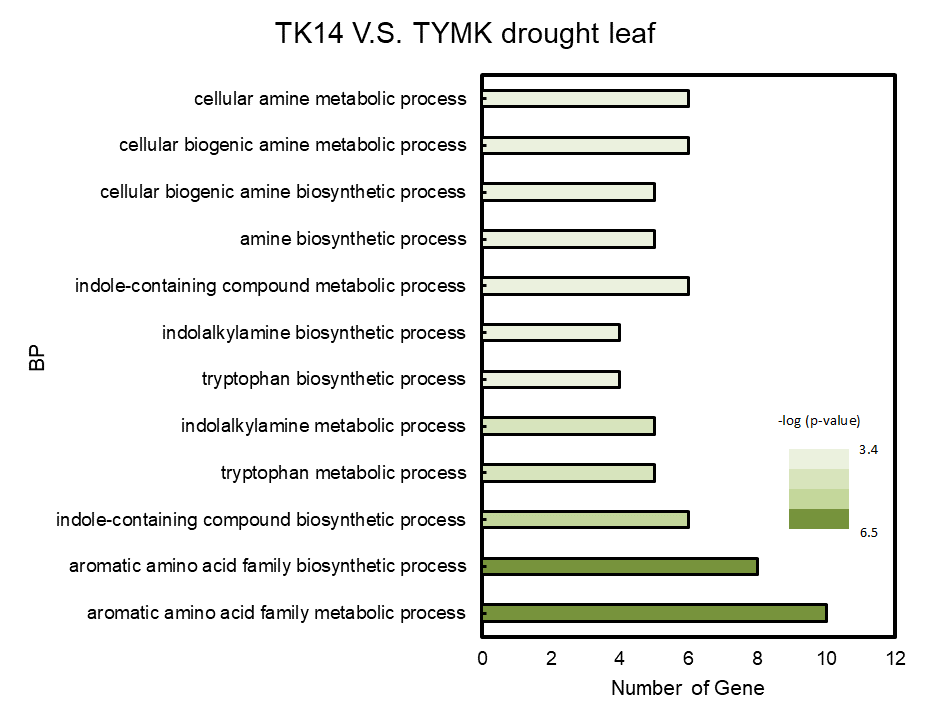
Supplementary Figure 4. Gene ontology categorization of DEGs between TK14 and TK14_12.1 under (A) control and (B) drought condition in leaf tissue. Gene with fold change ≥ 2 were defined as differentially expressed genes (DEGs) between TK14 and TK14_12.1 under control condition in leaf tissue.

(B)


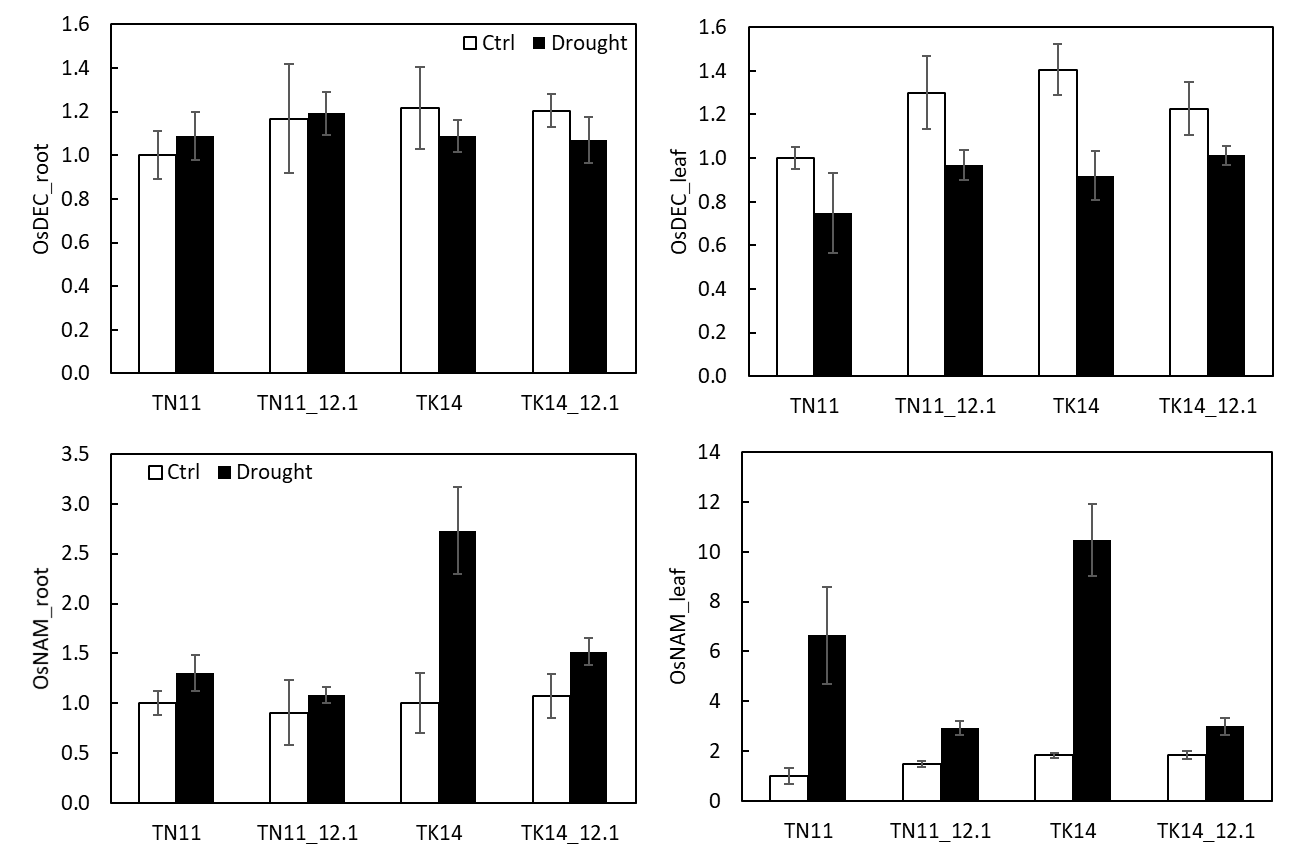


Supplementary Figure 5. Transcription of *OsDEC* and *OsNAM* in roots and leaves of TN11, TN11_12.1, TK14, and TK14_12.1 under control and drought conditions. Bars represent mean ± SE (n = 3). White and black bars indicate control and drought treatments, respectively.
